# Neurogenic mechanisms in bladder and bowel ageing

**DOI:** 10.1007/s10522-015-9554-3

**Published:** 2015-02-11

**Authors:** Richard N. Ranson, M. Jill Saffrey

**Affiliations:** 1 Department of Applied Sciences (Biomedical Sciences), Faculty of Health and Life Sciences, Northumbria University, Newcastle upon Tyne, NE1 8ST UK; 2 Department of Life, Health and Chemical Sciences, The Open University, Milton Keynes, UK

**Keywords:** Ageing, Bladder, Bowel, Anal sphincter, Incontinence, Constipation, Autonomic nervous system, Enteric nervous system

## Abstract

The prevalence of both urinary and faecal incontinence, and also chronic constipation, increases with ageing and these conditions have a major impact on the quality of life of the elderly. Management of bladder and bowel dysfunction in the elderly is currently far from ideal and also carries a significant financial burden. Understanding how these changes occur is thus a major priority in biogerontology. The functions of the bladder and terminal bowel are regulated by complex neuronal networks. In particular neurons of the spinal cord and peripheral ganglia play a key role in regulating micturition and defaecation reflexes as well as promoting continence. In this review we discuss the evidence for ageing-induced neuronal dysfunction that might predispose to neurogenic forms of incontinence in the elderly.

## Brief introduction and aims of this review

The lower abdomino-pelvic cavity contains organs including the small and large intestines, the bladder and components of the lower urinary tract. Numerous studies have demonstrated that these structures are subject to age-related changes that may lead to an increase in the incidence of bladder/bowel disorders in the elderly. The causes of these changes are likely to be multifactorial including ageing of the effector cells (e.g. smooth muscle) and also the cells that regulate their function (e.g. neurons of the central and peripheral nervous systems). The aim of this review is to discuss the potential neurogenic (e.g. defective neurotransmission) mechanisms resulting in ageing of these different abdomino-pelvic organs with an emphasis on the spinal and peripheral neurons that provide the efferent (motor) innervation of these structures to regulate bladder and bowel function.

## Age-associated bladder and lower bowel dysfunction in humans

Ageing of the bladder and lower bowel may result in problems of storage of faecal material and urine (waste), manifest in incontinence (Tariq et al. 2003; Wehrberger et al. 2012) and problems with the elimination of waste resulting in constipation or obstruction disorders (Rao and Go 2010). The prevalence of these conditions has been shown to increase with age in both sexes (Campbell et al. 1985; Kok et al. 1992; Teunissen et al. 2004; Irwin et al. 2006; Shaw et al. 2006; Collerton et al. 2009; Wehrberger et al. 2012). However it should be noted that many studies do not provide detailed information on types of dysfunction e.g. stress vs. urge incontinence; that studies have very different sample sizes and that sex/age-groups studied are not consistent. Furthermore, because of social stigma and embarrassment, it is likely that these conditions are under-reported by patients/sufferers.

Studies on urinary incontinence, in community based individuals, suggest prevalence rates to range from 11.6 % in individuals aged from 65 to 80 (Campbell et al. 1985) to 35 % in subjects aged 85+ (Wehrberger et al. 2012). This value increases to approximately 69 % in subjects aged 85+ confined to nursing homes (Xu and Kane 2013). Where studies distinguish between male and female sufferers (Collerton et al. 2009; Wehrberger et al. 2012) the incidence of urinary incontinence is found to be consistently higher in women aged 85+ e.g. 26.6–35 % compared to 12.6–24 % in men. Some studies have sought to differentiate between the types of urinary incontinence symptoms observed in the elderly. In a study of 19,165 men and women from five European countries, nocturia (the need to wake and pass urine at night) was found to be the most prevalent lower urinary tract disorder with 24 % of women and 21 % of men subject to more than two episodes per night (Irwin et al. 2006). This study also determined that the prevalence of overactive bladder (urge incontinence manifest by sudden uncontrolled contractions of the detrusor muscle) was 11.8 % in both sexes and that this figure increased with ageing. In very old patients aged 85+ the reported prevalence of overactive bladder was as high as 55/50 % in women and men (Wehrberger et al. 2012). Fewer, and less detailed, studies have determined the prevalence of faecal incontinence.

Constipation, faecal impaction and incontinence also increase in prevalence in the elderly, although as for urinary incontinence data are variable and collection methods are not consistent across studies. Figures for the prevalence of faecal incontinence, defined as one involuntary loss of faeces, range from as low as 3.1 % in a sample including both community and institutionalised adults (Campbell et al. 1985) to 50 % in a sample of patients in long-term care, aged 60+ (Chassagne et al. 1999). In contrast, figures for the prevalence of faecal incontinence in community (only) dwelling adults report it to be observed in 16.9 % of individuals aged 85+ (Kok et al. 1992). The higher prevalence of faecal incontinence in the institutionalised elderly has been associated with a history of urinary incontinence, constipation, poor mobility and neurogenic factors including poor cognitive function, dementia and spinal cord injury (Tobin and Brocklehurst 1986; Chassagne et al. 1999; Obokhare 2012). Where studies have distinguished between men and women (Collerton et al. 2009) the prevalence of faecal incontinence was found to be 9.3 % in women and 7.4 % in men aged 85 years+ in a sample consisting of both community dwelling and institutionalised elderly individuals. The higher incidence of both urinary and faecal incontinence in women may be due to pelvic floor muscle and nerve damage during childbirth (Kearney et al. 2006; Marsh et al. 2011; Mannella et al. 2013). There is also some data for the prevalence of double incontinence. In a postal questionnaire of 5,278 males and females 3 % of respondents reported that they suffered from both urinary and faecal incontinence (Teunissen et al. 2004).

Faecal incontinence secondary to constipation (or faecal impaction) and neurogenic incontinence have been suggested to be equally common in elderly individuals (aged 80+) in residential care with a study showing that in a small sample (less than a hundred individuals) of both men and women 52 % of patients developed faecal incontinence secondary to faecal impaction whilst in 48 % the incontinence was neurogenically mediated (Tobin and Brocklehurst 1986). Modern healthcare provision defines constipation as stool frequency of less than three bowel movements per week (Rao and Go 2010). Prevalence figures for constipation vary across studies. One large scale study (10,018 individuals) of adults aged 18+ suggested a prevalence rate of 14.7 % (Stewart et al. 1999). Prevalence rates are higher in the elderly. In community dwelling adults aged 65+ a self-reporting study suggested that around 40 % of individuals may have some form of constipation (Talley et al. 1996). In residential care, again, figures are variable. One study of long term nursing home residents (11,788) aged 80+ suggests prevalence figures of 10 % in both males and females from data obtained by examining medical records. By contrast reports that 74 % of nursing home residents used daily laxatives (Talley et al. 1996) suggest that the prevalence of constipation in residential care may be much higher. The aetiology of constipation may be variable with a number of potential causes suggested including metabolic factors (e.g. hypercalcaemia), diet (inadequate fluid/fibre), some medications (e.g. anti-cholinergic, calcium blockers) and neurogenic causes such as spinal cord injury (loss of autonomic regulation of the bowel) or loss of enteric neurons (Obokhare 2012; Rayner and Horowitz 2013; Coggrave et al. 2014). Chronic constipation can lead to faecal impaction (Rao and Go 2010) defined as the inability to evacuate large, hardened, dry, stools commonly lodged in the rectum (Obokhare 2012). Faecal impaction is common in the elderly e.g. of 460 patients admitted to UK hospital geriatric wards, over the course of one year, 196 (42 %) had faecal impaction (Read et al. 1985). Faecal impaction has been linked with an increased potential for future morbidity in impacted patients (Araghizadeh 2005).

## Psychological, lifestyle and financial consequences of bladder and bowel dysfunction in the elderly

Incontinence can have a detrimental effect on psychological well-being that contributes to an overall impact on the quality of life of elderly sufferers (Wald et al. 2007; Farage et al. 2008; Huang et al. 2010; Markland et al. 2010; Yip et al. 2013). The burden of suffering from urinary or faecal incontinence has been linked to symptoms of anxiety and depression (Coyne et al. 2012; Molinuevo and Batista-Miranda 2012). Anxiety symptoms could result from the perceived fear of having a bladder or bowel accident (leakage) that might prove to be socially embarrassing (Cartwright et al. 2011; Ness 2012). Initially, bladder/bowel incontinence results in the sufferer adopting complex coping strategies such as toilet mapping and/or food and fluid restriction (Srikrishna et al. 2009; Ness 2012). Over time incontinence leads to a curtailment of daily activities e.g. going out, shopping etc. which decreases self-esteem (Cartwright et al. 2011) and promotes social isolation (Yip et al. 2013). The most serious consequence of both urinary and faecal incontinence is a link to an increased risk of death in both community dwelling adults and those confined to nursing homes e.g. the ten month mortality rate in faecally incontinent patients in a nursing home was 26 % compared to 6.7 % for individuals in the continent control group (Chassagne et al. 1999; Nakanishi et al. 1999). Less information is available on the effects of constipation on lifestyle and mortality in the aged however these observations suggest that it plays a significant role. In a study of 2,870 males and females (aged 16–75+) across seven countries, including the UK, constipated individuals reported that it placed a significant burden on their quality of life (Wald et al. 2007). Both physical components (e.g. levels of pain, general health) and mental components (e.g. vitality, social function and mental health) were significantly impaired in more elderly sufferers and in particular in women. Mortality rates have also been found to be increased in community dwelling elderly women (aged 70–75) who persistently reported suffering from constipation (11 % mortality rate over 15 years) in comparison to those who were constipation free [8.2 %; (Koloski et al. 2013)].

The impact of bladder and bowel dysfunction on lifestyle, and quality of life in the aged also has a financial consequence. 2005 figures for assessing the costs of overactive bladder with urge urinary incontinence in the UK estimated total national costs to be 595 million euro/year with the average cost to a UK patient of 528 euro per annum for costs including e.g. prescription medication, including anti-anxiety/depressant medication, and incontinence pad use but not including costs for nursing home care (Irwin et al. 2009). Equivalent data for faecal incontinence costs in the UK are currently not available but figures from United States suggest an annual total cost of 4,110 dollars (~3,040 euro) per person per annum (Xu et al. 2012). Likewise, constipation has a financial cost. One more recent study (Taylor and Guest 2010) with data collected in 2007/2008 in a UK hospital suggests that the average cost of treating a patient (with a mean age of 65.6 years), suffering from chronic constipation, with the laxative Lactulose was approximately 510 euro (£420) over a six month course of treatment.

## Basic structure of the urinary system and terminal bowel

The bladder and bowel consist of smooth muscle with an inner lining of specialised and functionally important epithelial cells, with additional cell types that include nervous and vascular supplies, connective tissue cells, interstitial cells and immune system cells (which in the gut, constitute a vast population of diverse cells) but the properties and arrangement of the cells in these organ systems is very different. Here the main focus of this review is on the neuronal control of the bladder, bowel and lower urinary tract structures and a detailed discussion of other cell types is not included.

### The anatomy and cellular organisation of the bladder and urethra

The bladder is a hollow muscular organ connected to the kidneys by the ureters. Urine is excreted from the kidneys, passes through the ureters, and is stored in the bladder before elimination via the urethra during the micturition reflex (Lukacz et al. 2011). During periods of bladder filling the storage of urine is promoted by the actions of the internal and external urethral sphincters and pelvic floor musculature. During the micturition reflex these sphincters relax and the smooth muscle of the bladder (the detrusor muscle) contacts, resulting in the expulsion of urine (Lukacz et al. 2011). The bladder and urethra consist of smooth muscle with a specialised epithelial lining, known as the urothelium (Drake 2007; Birder et al. 2010a, b), but in comparison to that of the GI system, the smooth muscle of the bladder is less organised, reflecting its more limited role in storage of urine (compared with the role of GI smooth muscle in the mixing and movement of gut contents). Another major difference between the bladder and the GI system is that intramural neurons are not present in the bladder of all species e.g. they are numerous in guinea-pigs and in humans, less prevalent in cats, ferrets and rabbits and absent in rats (Mizuno et al. 2007). When present these intramural neurons are grouped into very small ganglia (Saffrey et al. 1992). In contrast to GI innervation, the major innervation of the bladder is from extrinsic neurons located outside the bladder wall.

### Bladder interstitial cells

Interstitial cells (ICs, cells with a similar morphology to fibroblasts, but which are implicated in the regulation of smooth muscle contractility) are also present in the bladder and urethra (McCloskey 2010, 2013). ICs are divided into subpopulations of cells within the different layers of the bladder wall and these subpopulations express different markers, including c-Kit, vimentin and platelet-derived growth factor receptor alpha [PDGFRα; (McCloskey 2010, 2013)]. ICs form close contacts with other ICs and also with nerve fibres and smooth muscle cells, and ICs are present in the lamina propria (the region of the bladder mucosa that lies between the urothelium and the detrusor muscle). Recent evidence has demonstrated that these ICs are functionally innervated (Gray et al. 2013). Thus, as in the gut ICs appear to play an intermediary role in the nervous control of the cells of the bladder wall. The function of ICs in the bladder is currently an area of much research interest, because recent evidence has shown altered IC function, including alterations in signalling properties and transformations in structure to a more fibroblast-like phenotype in bladder diseases including urgency and painful bladder syndrome (McCloskey 2013).

### Urothelial cells

The role of urothelial cells in bladder function is somewhat clearer; they respond to physical and chemical stimuli, and release neuroactive mediators, including ATP, nitric oxide (NO) acetylcholine (Ach) and Substance P (Birder et al. 2010b; Birder and Andersson 2013) that have the potential to modulate sensory nerve endings that course through the urothelium (Birder and Andersson 2013; Birder 2013). Finally, it has recently been proposed that the lamina propria may also play an important role in bladder function (Andersson and McCloskey 2014). In short, although much remains to be learned about the regulation of bladder function, it is now established that the interactions between innervation, ICs and urothelial cells are complex and that regulation of bladder function depends upon integration of their individual activities, hence there is significant potential for breakdown of bladder function during ageing.

### Innervation of the bladder and urethral sphincters

The bladder receives indirect innervation from parasympathetic autonomic neurons in the sacral parasympathetic nucleus of the spinal cord, which project via the pelvic nerve to the pelvic ganglia. Sympathetic innervation is also indirect and arises from neurons in the thoracolumbar spinal cord, which project via the hypogastric and pelvic nerves to the hypogastric ganglia/pelvic ganglia and ganglia of the lumbosacral sympathetic chain. Hence the body of the bladder is directly innervated by efferent fibres that arise from parasympathetic postganglionic neurons in the pelvic ganglia and intramural ganglia and by efferent fibres that arise from sympathetic postganglionic neurons in lumbosacral sympathetic chain and hypogastric ganglia/pelvic ganglia (de Groat 2006; Michel and Barendrecht 2008; Birder et al. 2010a; de Groat and Wickens 2013; Ochodnicky et al. 2013). The internal urethral sphincter receives innervation from the hypogastric and pelvic ganglia as described above for the bladder detrusor muscle. The external urethral sphincter is directly innervated by motor neurons in the sacral segments of the spinal cord (see next section).

In small rodents such as rats and mice the majority of the postganglionic neurons that supply the pelvic viscera are located in the major pelvic ganglion (MPG), which is equivalent to the more diffuse plexus of postganglionic cells supplying the pelvic organs of larger mammals (Santer et al. 2002). Furthermore the MPG in these animals contains groups of both parasympathetic and sympathetic postganglionic neurons making it a useful model to compare age-related changes in the efferent outflows to the pelvic viscera (Warburton and Santer 1993, 1994; Santer et al. 2002; Dowling et al. 2006). The parasympathetic fibres that regulate bladder function release acetylcholine and stimulate muscle contraction, whereas the sympathetic fibres stimulate relaxation by noradrenergic signalling. In addition to acetylcholine and noradrenaline, the efferent autonomic neurons that regulate detrusor smooth muscle contractility utilise a range of other neurotransmitters; e.g., ATP, NO, and neuropeptides (see above reviews). Efferent fibres also supply the urothelium.

Neuronal tracing and ultrastructural studies have shown that sensory nerve fibres originating in lumbosacral dorsal root ganglion (DRG), are present in the detrusor muscle, but there is also a significant innervation of the lamina propria lying next to the urothelium [see above and (Birder 2013)]. These fibres express a number of different neurotransmitters including the neuropeptides substance P and calcitonin gene-related peptide, CGRP (Gabella and Davis 1998).

### Spinal cord neurons that regulate bladder function

**Fig. 1:**
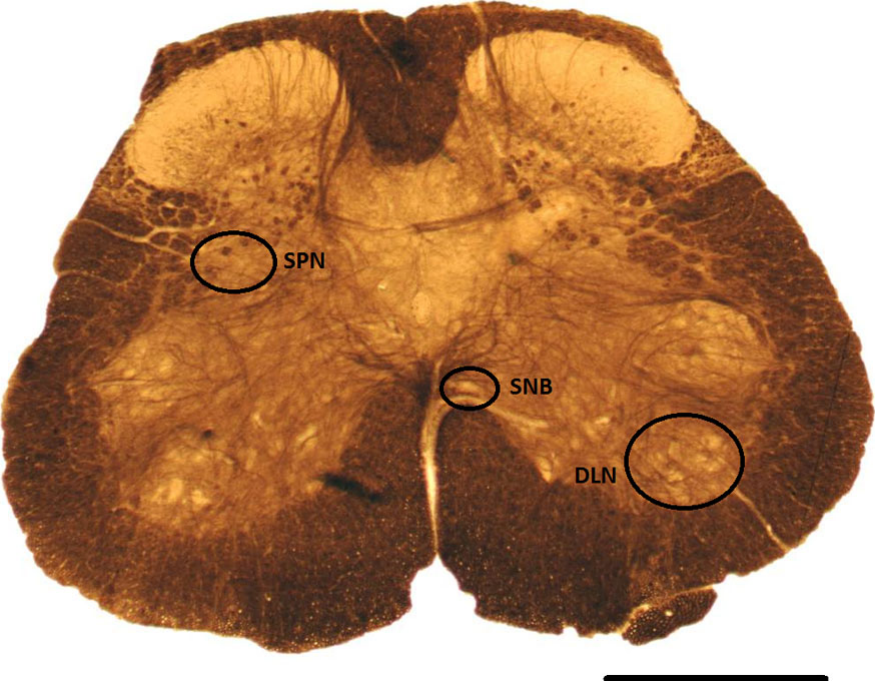
A 45 μm section of osmicated mouse spinal cord taken from spinal levels L5–S1. At this level groups of neurons that provide the parasympathetic innervation of bladder and bowel are located in the sacral parasympathetic nucleus (SPN). Sited more ventrally are motoneurons contained within the dorsolateral nucleus (DLN) and spinal nucleus of the bulbospongiosus (SNB) that provide axons to the pudendal nerve. A proportion of motoneurons in the DLN/SNB provide innervation of the external anal and urethral sphincters and the levator ani muscle and are involved in maintaining urinary and faecal continence. *Scale bar* 250 μm

A group of sympathetic preganglionic neurons, located within the intermediolateral cell column and dorsal grey commissure of upper thoracolumbar (L1–L2 rodents, T12–L2 humans) spinal cord (Ranson et al. 2006), activate the efferent pathways that promote urinary storage by relaxing bladder detrusor muscle and contracting the internal urethral sphincter (de Groat and Wickens 2013). At lumbosacral sacral levels (L5–S1 rodents, S2–S4 humans) a group of parasympathetic preganglionic neurons are sited in lamina VII in a region termed the sacral parasympathetic nucleus- see Fig. 1. (Ranson et al. 2006). Activation of these neurons promotes detrusor contraction and bladder emptying (Chai and Steers 1996; Blok 2002, de Groat and Wickens 2013). At lumbosacral spinal levels a somatic nucleus, termed Onuf’s nucleus in humans, contains motoneurons that provide innervation of the external urethral sphincter via the pudendal nerve (de Groat and Wickens 2013). During bladder filling these neurons are tonically active and promote the contraction of the external urethral sphincter thus preventing leakage of urine from the bladder (Thor and de Groat 2010). In rodents, structures homologous to Onuf’s nucleus consist of the dorsolateral nucleus (DLN) and spinal nucleus of the bulbospongiosus (SNB)—see Fig. 1. More specifically the DLN of rodents, via the pudendal nerve, projects to the ischiocavernosus (involved in anal flexion and reproductive reflexes) and external urethral sphincter muscles whilst the SNB provides innervation of the ventral bulbospongiosus (primarily involved in sexual reflexes), levator ani and external anal sphincters (Schroder 1980; Jordan et al. 1982; McKenna and Nadelhaft 1986; Ranson et al. 2007b, 2012).

### Brain and spinal interactions in the control of micturition

Micturition takes place as the result of a combination of voluntary control and involuntary reflexes. Urine is normally held in the bladder involuntarily, by the combined relaxation of the detrusor muscle and contraction of the internal and external urethral sphincters. Bladder filling stimulates sensory receptors within the bladder wall that respond to stretch. In mammals, including cats and rats, these receptors send information via myelinated A-delta fibres (coursing through the pelvic nerve) to the lumbosacral spinal cord (Birder et al. 2010a; de Groat and Wickens 2013). Afferent projections from the bladder terminate within the dorsal horn and collateral projections are also observed in the dorsal grey commissure (Rexed lamina X) and in the sacral parasympathetic nucleus; the site of parasympathetic neurons that modulate detrusor contraction (de Groat and Wickens 2013). In humans, sensory information is also relayed to a variety of nuclei in the brain, shown by functional imaging to be active during micturition behaviours (Griffiths and Fowler 2013). The CNS structures involved in micturition control have been extensively reviewed (de Groat 1998; Griffiths et al. 2009; Drake et al. 2010; Griffiths and Fowler 2013; Michels et al. 2014) however a short description of these is included here to provide context for later discussions on ageing and spinal control of lower urinary tract function.

Additional sensory information from the bladder is transmitted to the periaqueductal grey nucleus which provides connectivity to the pontine micturition centre (PMC). Sensory information is also relayed via the thalamus to other brain regions including the hypothalamus and areas of the forebrain such as the insula and pre-frontal cortex (Griffiths and Fowler 2013). It is thought that the insula provides interoception of bladder filling (Michels et al. 2014) whilst the pre-frontal cortex modulates voluntary control of the bladder and e.g. prevents inappropriate emptying of the bladder during social situations (Griffiths and Fowler 2013; Michels et al. 2014). For more comprehensive reviews of these afferent pathways see e.g. (de Groat and Yoshimura 2009; Griffiths and Fowler 2013).

Efferent control of the bladder is dependent on intact pathways that connect the PMC to the spinal cord, since transection of the brain at or below the level of the PMC abolishes micturition reflexes (Kuru 1965; Satoh et al. 1978). Neuronal tracing has demonstrated direct projections from the PMC to the intermediolateral cell column and sacral parasympathetic nucleus, which contain preganglionic neurons that influence bladder activity (Nuding and Nadelhaft 1998; Guo et al. 2013) and electrophysiological studies have identified cells in the PMC that are silent in the absence of bladder activity but fire prior to and during reflex bladder contraction (de Groat and Wickens 2013). PMC neurons have also been shown to project to spinal interneurons that subsequently project to cells in Onuf’s nucleus and thus the PMC has the capacity to modulate external urethral sphincter activity as well as bladder contractions (Blok 2002). It has been shown that in the region of 30–40 % of spinal projecting neurons in the PMC are corticotrophin releasing factor immunopositive (Valentino et al. 1995). Another neurotransmitter candidate for long descending pathways from the PMC, to the spinal cord, is glutamate, because the application of glutamate receptor antagonists in the region of the L6–S1 spinal cord inhibited contractions resulting from direct electrical stimulation of PMC cells (Matsumoto et al. 1995; de Groat 1998). Furthermore glutamate containing terminals have been demonstrated to make synaptic contact with spinal preganglionic neurons that project to the major pelvic ganglion (Santer et al. 2002).

As well as directly influencing spinal micturition control the PMC has been shown in rats to contain a significant projection to the locus coeruleus (Valentino et al. 1996). The locus coeruleus, along with the A5 adrenergic cell groups contain noradrenaline synthesizing cells that have been shown to project to both forebrain and spinal regions that are involved in the control of micturition reflexes (Fritschy et al. 1987; Lyons et al. 1989; Valentino et al. 1996). The locus coeruleus is thought to project primarily to the dorsal horn and intermediate regions of the spinal cord whilst the A5 cell group projects primarily to the ventral horn (Lyons et al. 1989). Other immunocytochemical studies have shown robust staining of catecholamine containing terminals in Onuf’s nucleus (and its rat homologues) as well as in the intermediolateral cell column where they are closely apposed to both parasympathetic and sympathetic preganglionic neurons (Ranson et al. 2003a, b, 2005b, 2006). These noradrenergic projections are likely to influence micturition behaviours since chemical lesioning of the locus coeruleus (in cats) results in hypoactive bladders (Yoshimura et al. 1990). Furthermore by acting on external urethral sphincter projecting motoneurons, adrenergic agonists have been shown to facilitate storage reflexes (Thor 2003; Birder et al. 2010a). Other medullary-pontine areas with notable projections to the lumbosacral cord include the medullary raphe nuclei which provide serotoninergic/substance P containing projections to bladder innervating preganglionic cells and urethral sphincter innervating motoneurons (Santer et al. 2002; Ranson et al. 2003a, 2005a, b, 2006). Serotoninergic effects on voiding behaviour including micturition reflexes and urethral sphincter activity differ depending on species studied e.g. in cats serotonin inhibits parasympathetic outflow to the bladder whilst the opposite is seen in rats accompanied by sphincter excitation (de Groat 2002).

## Effects of ageing on the urinary system

### Bladder-functional changes

While changes in bladder function during ageing are known to occur in humans, and have also been described in rats, guinea-pigs and mice, relatively few studies have been performed and much remains to be understood about how ageing affects bladder function (Nordling 2002; Siroky 2004; Smith 2010). Moreover some data are conflicting. In the rat, age-related reductions in muscarinic responses have been described (Zhao et al. 2010). In guinea-pigs, contractile responses to electrical field stimulation (EFS) were impaired in aged animals (Gomez-Pinilla et al. 2007). The cholinergic component of responses to EFS were maintained, but the purinergic component was reduced in ageing guinea-pigs (Gomez-Pinilla et al. 2007). Excitatory sensory actions on bladder contractility were also reduced in ageing animals in this study. Reduced levels of components of signal transduction pathways in smooth muscle cells have also been described in the guinea-pig bladder (Gomez-Pinilla et al. 2008). In ageing mice, physiological studies indicate that smooth muscle responses to cholinergic and purinergic stimulation (Lagou et al. 2006; Gomez-Pinilla et al. 2011b) and calcium signalling (Gomez-Pinilla et al. 2011b) are impaired. Spontaneous contractile activity, a prominent feature in the bladder of young animals, is markedly reduced in older animals (Lagou et al. 2006). Similar changes have been described in overactive bladder syndrome (Drake et al. 2005). A more recent study also suggests that sensory mechanisms are impaired in the ageing mouse bladder (Smith et al. 2012). This study employing a urethane-anesthetized mouse cystometry model found that intervoid intervals, voiding volumes, and flow rate of voiding all increased with age. Furthermore, calculations, employing substitute (indirect) measures of mechanotransduction, to approximate wall stress during filling suggested a loss of bladder volume sensitivity with increasing age in the C57BL6 mouse.

### Bladder-changes in structure and innervation

Studies at the light microscope (LM) level have shown structural changes in ageing rat bladder (Zhao et al. 2010). An increase in collagen content and a reduction in both muscle mass and urothelial thickness in aged animals were reported by these authors. More recent work in aged mice (24 months) has shown that there are raised levels of purinergic receptor expression in the urothelium that has been linked to bladder overactivity (Daly et al. 2014). Furthermore, parallel studies have shown that increased levels of oxidative stress can produce an upregulation of TRPM8 (Transient receptor potential cation channel subfamily M member 8) receptors in the urothelium of aged animals and that this may affect calcium signalling in this tissue (Nocchi et al. 2014). These observations, though not the main focus of this review suggest that further study of urothelial-afferent interactions, in bladders of the elderly (and aged animal models) may provide important new insights into lower urinary tract dysfunction.

Ultrastructural analysis of the ageing bladder is limited to only few studies, which have not provided a comprehensive description of changes across the bladder wall and in different cell types. In elderly humans (65–91 years) Elbadawi (Elbadawi et al. 1993, 1998) described ageing in the detrusor and observed apparent smooth muscle atrophy manifest by vacuolated sarcoplasm, myofilament disarray and sarcolemmae depleted of caveolae. Furthermore, throughout the detrusor muscle neuronal processes showed signs of degeneration with depleted synaptic vesicles. Collagen fibre accumulation was also reported. In rodents ultrastructural data on bladder ageing is also scant. In rats a loss of smooth muscle caveolae has also been reported (Lowalekar et al. 2012) however the oldest age examined was a 12 month rat (early middle age). A further study has reported changes in the urothelium and presented evidence for lipofuscin accumulation in urothelial cells of ageing mice (Perse et al. 2013).

Structural studies at the LM level have demonstrated changes in the sympathetic innervation of the bladder in ageing rats. A reduction in sympathetic fibres in the bladder body of 24 month compared to 3 month old rats has been reported (Warburton and Santer 1994). A reduced density of CGRP-, SP- and nNOS-immunoreactive sensory nerve fibres in the bladder of 24 month compared to 3 month old rats and a reduction in the number of DRG neurons expressing these mediators in old rats have also been described (Mohammed and Santer 2001, 2002). Intramural neurons may also be affected by ageing; a reduction in the number of intramural neurons in the ageing guinea-pig bladder has been reported (Mizuno et al. 2007), although changes in their processes have not been studied in ageing animals. Equivalent structural data on aged bladder for other species including, mice and humans, appears lacking and given the impact of age-related bladder dysfunction (described above) there is a pressing need for studies to be carried out in pursuit of this information.

## Anatomy and cellular organisation of the terminal bowel

The region of the terminal bowel that regulates the storage and expulsion of faeces is the ano-rectum. The rectum lies between the distal colon and anal canal. Faeces are stored in the rectum, and expelled through the anal canal by the actions of the anal sphincter complex (ASC). The rectum exhibits some differences from, but has the same general structure and organisation as the distal colon, while the ASC has some distinct anatomical features.

### The anatomy and cellular organisation of the rectum and anal sphincter complex

**Fig. 2:**
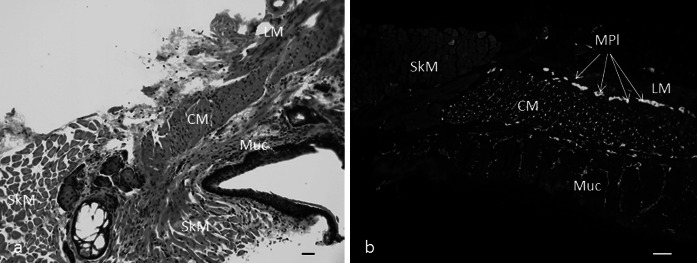
Anal sphincter region of mouse gut **a** stained with haematoxylin and eosin, and **b** immunolabelled with antiserum against the pan-neuronal marker PGP9.5. *CM* circular muscle (IAS), *LM* longitudinal muscle, *MPl* myenteric plexus, *Muc* mucosa, *SkM* skeletal muscle of the external anal sphincter. *Scale bars* represent 100 μm

Like that of the rest of the GI tract, the rectal wall consists of two outer smooth muscle layers (known as the muscularis externa, consisting of longitudinal and circular muscle layers), between which a complex network of ganglia, the myenteric plexus, is located. A second network of smaller ganglia, the submucous plexus, is present in the connective tissue that lies between the muscularis externa and the mucosa. These two networks of intrinsic ganglia constitute the enteric nervous system (ENS). The smooth muscle layers are richly supplied with nerve fibres, including intrinsic nerves and the processes of extrinsic fibres (see Furness 2006). An inner layer of smooth muscle, the muscularis mucosae, separates the submucosal connective tissue and mucosa in most regions of the gut. The general structure of the terminal bowel at the ASC is broadly similar to the rest of the GI tract, except that it has a specialised area of thickened smooth muscle, the internal anal sphincter (IAS, shown for mice in Fig. 2). Several areas of skeletal muscle lie around the IAS but outside the gut wall; these are the external anal sphincter (EAS) and the pelvic floor muscles (Raizada and Mittal 2008; Seney et al. 2009; Thor and de Groat 2010; Cobine et al. 2011; Ranson et al. 2012). In both humans and rodent models the sphincters provide a role in maintaining continence. However in rodents the muscles of the pelvic floor e.g. the levator ani are more sexually dimorphic than in humans, with greater muscle mass being associated with penile erection in the male of the species and little evidence that the levator ani plays a role in either sexual function or continence in females (Ranson et al. 2007b). The IAS is under involuntary control (see below) but contraction of the external sphincters and pelvic floor muscles is controlled consciously (Bajwa and Emmanuel 2009). The physiological mechanisms of defaecation are described below.

The IAS plays an important role maintaining faecal continence. In the normal resting state, the IAS is contracted and it has a basal tone. It is estimated that in humans the IAS contributes some 50–85 % of total anal sphincter tone (Lestar et al. 1989; Cook et al. 2001; Bharucha 2006, 2008), the remainder being contributed by the external anal sphincter (Krogh and Christensen 2009). The basal tone of the IAS is in large part due to inherent properties of the smooth muscle and to a lesser extent to extrinsic excitatory neural input by sympathetic nerves [see below, and (Cook et al. 2001; Bharucha 2006, 2008; Rattan and Singh 2011)].

### Bowel interstitial cells

Interstitial cells, in the gut known as interstitial cells of Cajal (ICCs), and the structurally similar fibroblast-like cells (FLCs) are present throughout the GI tract, including the terminal bowel. They are present around the myenteric ganglia (ICC-MY), in the muscle layers (ICC-LM and ICC-LM), and in some gut regions, in the submucosa see e.g. (Komuro 2006). In the AS region, the density of ICC-MY has been reported to be reduced compared to other GI regions, but intramuscular ICC are abundant in the IAS (Duffy et al. 2012). ICCs are c-Kit positive and play a key role in the regulation of smooth muscle contraction and relaxation (Sanders et al. 2010). FLCs are PDGFR-α positive and have also now been implicated in the regulation of smooth muscle contractility [e.g. (Cobine et al. 2011)].

### The intestinal mucosa

The intestinal mucosa consists of the epithelium, which is composed of diverse epithelial cells (including stem cells, enteroendocrine cells (EECs) that release gut peptide hormones or serotonin, absorptive enterocytes and mucus-secreting goblet cells) and underlying connective tissue that is richly supplied with nerve fibres, associated glial cells, blood vessels and immune system cells. The intestinal mucosa is thus a highly complex tissue with a range of important roles, including barrier function and defence, as well as absorption of nutrients and also neural and endocrine signalling and appetite regulation. It is also the interface with the microbiota, which are now appreciated to have a major influence on the whole organism.

There is evidence that changes in mucosal epithelial cells take place during ageing. For example, some populations of gut hormone-producing EECs have been found to increase in number, while others decrease in old age [reviewed in (Saffrey 2014)]. Elucidating these changes is important as appetite is often reduced in the elderly. Recent evidence has demonstrated abnormal tight junctions and reduced expression of tight junctional proteins and mRNA in the ageing mouse small intestine (Ren et al. 2014), and impaired permeability and reductions in tight junction proteins in the colon of aging baboons (Tran and Greenwood-Van Meerveld 2013). Changes in the immune system (Mabbott et al. 2014; Man et al. 2014) and the microbiota (Biagi et al. 2012; Claesson et al. 2012) during ageing have also been described. The interaction between the microbiota, the cells of the mucosa (including enteric nerves), and the central nervous system is thus of key importance in health and in ageing and should be a major area for future research.

### Innervation of the ano-rectum

Like other parts of the digestive tract, the ano-rectum is innervated by intrinsic neurons of the ENS, and by extrinsic sensory and autonomic neurons (Furness 2006, 2012). The ENS is both the largest and the most complex division of the peripheral autonomic nervous system (Furness 2006, 2012). Enteric neurons are very numerous and functionally diverse, including intrinsic sensory neurons, interneurons and different types of motoneurons that regulate the functions of smooth muscle, intestinal blood vessels and the epithelium, and also intestinofugal neurons. Some enteric neurons form close associations with immune system cells, which are predominantly, but not exclusively located in the mucosa (Vulchanova et al. 2007; Phillips and Powley 2012). The ENS also contains specialised glial cells, which differ from the satellite cells found in other peripheral ganglia, but show similarities with CNS astrocytes (Ruhl 2005). Recent evidence shows that enteric glia exhibit different properties in different layers of the gut wall [e.g. in the myenteric plexus, smooth muscle and mucosa (Gulbransen and Sharkey 2012)]. It has also been shown that they interact with both neurons and non-neuronal cells such as intestinal epithelial cells and have a range of important roles in GI functions (Gulbransen and Sharkey 2012). For example, enteric glial cells have been shown, together with enteric nerves and epithelial cells acting as a “digestive neuronal-glial-epithelial unit”, to regulate barrier function (Neunlist et al. 2013), to express neurotransmitter receptors, and synthesise transmitter precursor molecules, so are likely to be involved in modulation of enteric neurotransmission (Gulbransen and Sharkey 2012) and to have a neuroprotective function (De Giorgio et al. 2012).

The terminal bowel (but not the EAS) receives postganglionic parasympathetic and sympathetic innervation from neurons of the pelvic ganglia. As is the case for the innervation of the bladder, the ganglionic organisation of the peripheral neurons that innervate the terminal bowel varies between species. The preganglionic input to sympathetic neurons is from the lumbar spinal cord; that of the parasympathetic is from the sacral spinal cord see e.g. (Keast 2006). The sensory innervation of the terminal bowel arises from the thoracolumbar spinal cord, via splanchnic nerves, and from the lumbosacral spinal cord, via pelvic nerves.

### Control of defaecation

Normal defaecation depends upon the voluntary relaxation of the EAS and pelvic floor muscles and the involuntary relaxation of the IAS. This involuntary action is the recto-anal inhibitory reflex (RAIR). The RAIR is stimulated when stools pass into the rectum from the sigmoid colon and cause rectal distension. This distension is sensed by mechanoreceptors in the rectal wall, which cause a neurally-mediated relaxation of the IAS muscle. The reflex is an intrinsic one, as it occurs even after spinal cord transection and is absent in patients with Hirschprung’s disease, in which the terminal bowel lacks enteric neurons (Cook et al. 2001; Bharucha 2006, 2008).

Like other intestinal reflexes, the RAIR involves intrinsic sensory and motoneurons. Relatively little is known of the properties of the intrinsic sensory neurons in the rectum. In other parts of the gut intrinsic sensory neurons have been much studied, and include neurons that respond to distension, mechanical stimuli and chemical changes, including serotonin, released from enterochromaffin cells [see (Bertrand and Thomas 2004)]. Much more is known about the intrinsic motoneurons that mediate relaxation of the IAS smooth muscle. These neurons are non-cholinergic, non-adrenergic (NANC) neurons and there is good evidence that they use nitric oxide (NO) (Rattan et al. 2005; Terauchi et al. 2005). However, recent evidence indicates that other inhibitory substances including ATP, VIP and carbon monoxide are also involved (McDonnell et al. 2008) and that NANC excitatory neurons may also play a role in IAS contraction, at least in the mouse (Keef et al. 2013).

ICCs have also been shown to have a role in the regulation of the RAIR e.g. (de Lorijn et al. 2005). FLCs are also present and are closely associated with the inhibitory nerves and ICCs in the smooth muscle of the IAS (Cobine et al. 2011), suggesting that they, too may play a role in the RIAR and hence in regulation of defaecation.

### CNS control of intestinal functions

As already described, the major innervation of the GI tract is intrinsic, from neurons of the ENS. Extrinsic efferents, however, play a role in modifying gut functions, and are responsible for the voluntary control of defaecation, by regulating the activity of the EAS and the pelvic floor muscles.

Extrinsic parasympathetic efferents reach the entire GI tract via two main routes. The proximal GI tract (as far as the proximal-mid colon) receives extrinsic parasympathetic efferent innervation via the vagus nerve (Mourad and Saade 2011). Since this review is focused on the terminal bowel, we do not discuss these pathways any further here. Extrinsic parasympathetic efferents to the distal bowel arise from spinal segments S2–S4 in humans and innervate the distal colon, anorectum and internal anal sphincter. These pathways modulate peristalsis and are involved in the defaecation reflex. The sympathetic innervation of the distal bowel originates in spinal segments T5–L2 and these thoracolumbar outflows inhibit bowel movement and maintain faecal continence by contracting the internal anal sphincter (Winge et al. 2003). The thoracolumbar nerves are mixed motor/sensory and sensory information is transmitted by afferent fibres, with cell bodies located in dorsal root ganglia immediately peripheral, to the T5–L2 spinal cord. (Phillips et al. 2010; Mourad and Saade 2011). The motoneurons that regulate the voluntary response of the EAS and pelvic floor muscles during defaecation are located in the sacral spinal cord.

Alongside spinal/peripheral nerves that regulate bowel function there is increasing evidence of higher CNS regulation of gastrointestinal structures. Notable structures that have been identified include the limbic system, the hypothalamus and the periaqueductal grey which are brain regions associated with homeostatic regulation, pain processing and emotional behaviours. A comprehensive discussion of these is provided in (Jones et al. 2006; Drake et al. 2010).

## Effects of ageing on the lower bowel

### Ano-rectum-functional changes

Despite the increase in constipation, faecal incontinence and impaction seen in the elderly population, relatively few studies have analysed these changes in detail, and animal models have been little used. Those studies that have been performed on ageing gut have provided conflicting results on changes in responses to nerve stimulation and applied agonists (Hoyle and Saffrey 2012), possibly due to differences in the techniques used and the ages and strains of animals studied. There is, however, evidence that gastrointestinal transit is impaired in animal models and recent evidence (Patel et al. 2014) has shown that not only is colonic transit impaired in ageing mice, but the frequency of stool production is also decreased. A reduction in faecal output has also been described in ageing rats (Smits and Lefebvre 1996).

### Ano-rectum-structural changes

A number of studies have described changes in the gastrointestinal tract during ageing [see (Saffrey 2014)] but most work has focused on the intestines and stomach, very few studies have examined age-associated changes in the cells of the ano-rectum. In other parts of the gut, alterations in the properties of smooth muscle have been described in ageing. In rats, these include increased thickness of the smooth muscle layer (Hoyle and Saffrey 2012) and mitochondrial structural abnormalities (Lopes et al. 2004). Decreases in calcium currents and intracellular calcium stores have been described (Xiong et al. 1993) but increases in mitochondrial and sarcoplasmic reticulum calcium stores have also been reported (Lopes et al. 2006). Impaired signal transduction pathways have also been described in smooth muscle of ageing rat colon (Bitar 2003; Somara et al. 2007; Saffrey 2014). Reductions in the density of interstitial cells (Gomez-Pinilla et al. 2011a) and the ENS have been reported.

### Enteric nervous system changes

Changes in the enteric nervous system during ageing have been quite widely studied in a range of species (including humans) and gut regions (Phillips and Powley 2007; Bitar et al. 2011; Saffrey 2013) although the anal sphincter region has been very little studied. Many, but not all studies have reported a reduction in the number of neurons, particularly cholinergic neurons in aged animals, but comparison of the data is complicated by the different approaches for quantification of neuronal numbers used [see (Kapur 2013; Saffrey 2013)]. Recent studies that have standardised for gut growth and stretch of whole mounts have not detected a neuronal loss in 24 month-old mice, although evidence of neurodegeneration was seen (Gamage et al. 2013). However, it is also possible that animal husbandry may influence the observed effects of ageing on enteric neuronal loss; caloric restriction has been found to reduce myenteric neuronal losses in old rats (Thrasivoulou et al. 2006; da Silva Porto et al. 2012). Levels of reactive oxygen species (ROS) have been shown to be higher in myenteric neurons from old rats (Thrasivoulou et al. 2006) and mice (Jurk et al. 2012) than in those from young animals, and caloric restriction was shown to reduce the rate of ROS generation by myenteric neurons from rat ileum (Thrasivoulou et al. 2006).

Further information about the mechanisms of neuronal ageing in the ENS has come from a recent study demonstrating that enteric neurons in ageing mice exhibit a senescence-associated phenotype (Jurk et al. 2012). The senescence-associated phenotype has been described in a number of cell types and is induced in response to DNA damage. Cells exhibiting this phenotype have foci of DNA damage incorporating activated histones such as γH2A.X, elevated ROS levels, and express activated p38 MAP kinase, and also interleukin-6 and senescence-associated β-galactosidase activity (Jurk et al. 2012). The senescence-associated phenotype is pro-inflammatory, and has been proposed to lead not only to dysfunction of the cells that display it, but also, by secretion of inflammatory mediator, to damage of nearby cells (Nelson et al. 2012).

Alpha-synuclein and hyperphosphorylated Tau immunoreactive aggregates have also been shown to be present in ageing enteric neurons (Phillips et al. 2009). Age-related changes in the ENS are described in detail in (Saffrey 2013). In the ano-rectum recent work has described a reduction in the density of nNOS and Substance-P –immunorecative nerve fibre in the circular muscle of the ageing mouse IAS (Wang et al. 2013).

### Changes to extrinsic nerve fibres in the gut

To our knowledge, detailed studies of changes in the extrinsic innervation of the most terminal bowel region (the ano-rectum) during ageing have not yet been performed;  with work focusing mainly on the innervation of the stomach and small intestine, and to a lesser extent, on the colon. Here we briefly summarise the changes that have been reported in these areas, to highlight that this is likely to be an area of importance in the terminal bowel.

Analysis of changes in the density of extrinsic parasympathetic nerve fibres in the GI tract during ageing is difficult, because populations of intrinsic enteric neurons express the same markers as extrinsic parasympathetic nerves. A similar problem exists in many species for extrinsic afferent fibres. Studies of change to the extrinsic innervation have therefore involved anterograde tracing (Phillips and Powley 2001). Changes in the vagal innervation of the rat gut during have been studied in this way, but the vagus supplies only a minor part of the extrinsic innervation to the proximal parts of the large intestine, and no age-related changes were reported in this region or in the small intestine (Phillips and Powley 2001).

Sympathetic nerves are more readily studied, because very few enteric neurons express tyrosine hydroxylase, or contain catecholamines (visualised by glyoxylic acid fluorescence), hence these can be used as markers. A reduction in the density of varicosities and glyoxylic acid fluorescence has been described in the rat small intestine, along with swollen axons (Baker and Santer 1988). Swollen axons have also been seen in other studies, including recent work (Phillips et al. 2013) and are considered to be one marker of axonopathy. However, this recent detailed work has also shown a range of other age-associated changes, including discrete foci of hyperinnervation, suggesting complex changes that may result from local changes in the GI environment (Phillips et al. 2013).

Functional changes in the extrinsic innervation (pudendal nerve) to the anal sphincter in the elderly have been described (Hall 2002) which include an increased latency of pudendal nerve firing which may contribute to anal sphincter dysfunction. Further physiological and anatomical studies of age-related changes in the extrinsic innervation to the ASC and rectum are therefore clearly warranted.

## Interstitial cell ageing in the bowel and bladder

Interstitial cells are now known to play essential roles in the regulation of intestinal muscle contractility and most likely also in the bladder (McCloskey 2013). Changes in the density or properties of ICCs and FLCs during ageing, however, have been very little studied. A reduction in the density of c-Kit-immunoreactive ICCs has been described in the ageing human stomach and colon (Gomez-Pinilla et al. 2011a), the rat colon (Jo et al. 2014) and in the mouse distal colon, rectum and anal sphincter (Gamage et al., *In preparation*).

## Effects of ageing on extrinsic regulatory systems of bladder and bowel

### Ageing and peripheral ganglia

The major pelvic ganglion (MPG) in rodents has been the focus of a number of ageing studies and in the context of this review is important because it contains autonomic postganglionic neurons that project to both the bladder and terminal bowel (colon/rectum) (Keast et al. 1989, Luckensmeyer and Keast 1995). Early work (Partanen et al. 1980) on pelvic ganglia showed that catecholamine levels potentially decreased whilst the amount of lipofuscin increased in ganglion cells providing some of the first evidence that the MPG was subject to age-associated change. A subsequent investigation confirmed that lipofuscin accumulates in these cells but also importantly showed the presence of enlarged and vacuolated neurons—indicative of neurodegenerative change (Golomb et al. 2001). These potentially neurodegenerative changes may be largely confined to the sympathetic population of MPG cells since further studies by Santers’s group (Warburton and Santer 1993) demonstrated a 53 % decline in the number of tyrosine hydroxylase immunopositive cells—a marker of sympathetic cells in the MPG. Furthermore synaptic transmission to the sympathetic cells of the MPG is likely to be impaired in ageing, because Synapsin 1 staining of nerve terminals surrounding these cells decreases by 50 % (Warburton and Santer 1995). The cause of these degenerative changes with age is yet to be fully determined but impaired calcium regulation and signalling may play a role since the calcium binding protein Calbindin-D28K has been shown to significantly decrease with ageing in sympathetic MPG cells of rats (Corns et al. 2000). In the few other studies of aged MPG a decrease in the expression of brain nitric oxide synthase in the ganglion, no significant changes in purinergic P2X2 receptor expression and increased deposition of connective tissue have been reported (Warburton and Santer 1997, Salama et al. 2002; Dowling et al. 2006). Putting the current evidence together, the key observation, particularly from Santer’s work, is that there is a clear and selective attrition of sympathetic cells within the MPG whilst parasympathetic cells appear to remain largely unaffected by ageing in these studies (Santer et al. 2002).

These observations on changes in peripheral ganglion neurons are in keeping with the observations on reduced fibre density and degenerative changes in the ageing bladder and intestine. Although this review focusses predominantly on changes to the efferent motor networks of neurons modulating bladder and bowel function, it should be noted that there is some data to suggest that sensory afferent pathways may also be affected by ageing in aged rodents. Parallel studies looking at lumbosacral dorsal root ganglion neurons showed significant decreases in NOS immunoreactive profiles and in the numbers of dorsal root ganglion cells immunopositive for calcitonin gene related peptide and substance P in aged rats (Mohammed and Santer 2001, 2002). Additional observations have been made in dorsal root ganglion cells providing afferent information from the terminal bowel. The effects of ageing on these cells include a loss of Nissl material, the accumulation of lipofuscin and signs of pathology including inclusion bodies and protein aggregates (Phillips et al. 2010). These studies suggest that the afferent pathways subserving bladder/bowel function in rodents may also be perturbed by ageing.

### Ageing and spinal cord

Because the spinal cord contains both autonomic and somatic motoneurons that regulate bladder and bowel function (refer to previous sections) then studies of age-associated changes to these neurons may be key to understanding neurogenic forms of bladder and bowel dysfunction in the elderly. In a series of studies using quantitative immunocytochemistry a number of significant changes have been noted in terms of the innervation patterns contained within specific spinal nuclei that govern these behaviours. Similar to the relationship described for the MPG (see above), sympathetic preganglionic neurons and their innervation may be more prone to the effects of ageing. Studies have shown that sympathetic preganglionic neurons, projecting to the MPG, are reduced in length and exhibit less branching (Dering et al. 1996, 1998). In addition, when comparing the total number of glutamate immunoreactive boutons on the dendrites of these cells in young (3–4 months) and aged rats (24 months), in comparison to a similar sample of parasympathetic neurons, then a significant decline was seen only in the sympathetic population (Santer et al. 2002). Furthermore this study showed a significant reduction in chemically unidentified synaptic inputs to these cells. Subsequent studies looking at the density of immunocytochemically identified terminals within the location of the IML, of rats, where these preganglionic are located (spinal segments L1–L2) indicate that some of these terminals might contain serotonin, substance P or catecholamines such as adrenaline and noradrenaline since immunocytochemical markers for these neurotransmitters all significantly decline within the intermediolateral cell column and dorsal grey commissure at L1–L2 levels (Ranson et al. 2003a, 2005a). Since the sympathetic preganglionics in L1–L2 spinal cord modulate bladder storage, such age-related declines may be of significance. By way of contrast, changes in innervation patterns associated with MPG projecting parasympathetic preganglionic neurons (associated with the micturition reflex) were more variable than those seen in the sympathetic regions. In the rat sacral parasympathetic nucleus the density of substance P and tyrosine hydroxylase significantly decreased between 3–4 and 24 months, but serotonin did not significantly decline (Ranson et al. 2003a, 2005a).

Age-related changes in the densities of immunoreactive terminals were also observed within the dorsolaterateral nucleus (homologue of Onuf’s nucleus in humans) of the rat, which contains somatic motoneurons that innervate the external urethral sphincter and ischiocavernosus muscles. Results were again variable with serotonin immunoreactive densities significantly decreased, substance P and tyrosine hydroxylase showing no change and vesicular acetylcholine transporter containing terminals significantly increased (Ranson et al. 2003a, 2005a, 2007a). Whilst the physiological significance of these observations is unclear, the results do confirm that the DLN is subject to neurogenic age-related changes which, could ultimately influence external urethral sphincter tone in these animals. In rats the pudendal nerve innervates the ventral bulbospongiosus, levator ani and external anal sphincters, of which the latter structures may play a role in faecal continence. In a study combining retrograde labelling of pudendal motoneurons innervating the levator ani, with immunocytochemistry to determine glutamate terminals, it was found that glutamate terminal innervation onto dendrites of these cells declined by 21 % (Ranson et al. 2007b).

One common factor for all of the age-related declines discussed above is that the neuronal inputs principally derive from long descending projections within the brain (see above). A loss of hind limb motor function has previously been linked to the neurodegenerative loss of serotonin releasing long-descending axons from the medulla to lumbosacral regions of the spinal cord that govern hind limb muscle contraction (Johnson et al. 1993). Alongside decreased serotoninergic axonal projections in the cord of aged rats it was also observed that some of the remaining serotonin fibres exhibited an aberrant morphology characteristic of neurodegeneration. Similar swollen axons were observed in spinal nuclei associated with bladder/bowel control (Ranson et al. 2003a). Together, the observations discussed here suggest that ageing also deleteriously affects spinal neurons that govern bladder/bowel reflexes and continence and that these changes may be linked to ageing of CNS neurons.

### CNS ageing

Very few studies have been carried out that have sought to determine any age-related CNS changes (outside of the spinal cord) that might influence bladder and bowel function in the elderly. Some recent work has employed functional magnetic resonance imaging to look at brain activation during micturition in humans with urge incontinence. These studies have found lower activation in structures including the insula and dorsal anterior cingulate cortex, leading the authors to conclude that with increasing age there are weaker signals in the bladder control network as a whole and that this may be responsible for the development of urge incontinence (Griffiths et al. 2009; Griffiths and Fowler 2013). Similar studies on ageing related bowel function are yet to be performed.

## Conclusions including other factors that may contribute to ageing of the urinary system and bowel

There is now considerable evidence that degenerative changes and other alterations such as decreased transmitter/receptor-related expression and/or impaired intracellular signalling (e.g. Ca^2+^ signalling) occur in some of the populations of neurons that regulate bladder and bowel function. Not all neuronal populations, however, seem to be similarly affected e.g. parasympathetic cells versus sympathetic cells in controlling bladder function. There is also variation in the extent of the changes seen in some populations of enteric neurons. The differences between different populations may in part be due to differences in the inherent properties of distinct groups of neurons. Differences between studies that have quantified neuronal losses have also emerged (see Saffrey 2013). Additionally the variable reports of the effects of ageing on bladder/bowel neurons may be the result of differences in the strains or even animal colonies being studied, or how they are maintained. There is evidence, for example, that diet may influence age-related changes in enteric neurons.

A number of other factors are likely to impact on changes in neurogenic mechanisms during ageing of the urinary system and bowel. These include cells of the immune system, which play different but important roles in both the bladder (e.g. during urinary infections), and bowel, and the microbiota. The gut microbiota are now appreciated to play a major role in many aspects of the biology of the whole organism, including the gut-brain axis, and recent evidence indicates that changes in the intestinal microbiota occur during ageing (see Saffrey 2014). These aspects will be important in future studies of regulation of bladder and bowel function during ageing.
